# HTRA1 in Placental Cell Models: A Possible Role in Preeclampsia

**DOI:** 10.3390/cimb45050246

**Published:** 2023-05-01

**Authors:** Giovanni Tossetta, Sonia Fantone, Stefano Raffaele Giannubilo, Andrea Ciavattini, Martina Senzacqua, Andrea Frontini, Daniela Marzioni

**Affiliations:** 1 Department of Experimental and Clinical Medicine, Università Politecnica delle Marche, 60126 Ancona, Italy; s.fantone@pm.univpm.it (S.F.); m.senzacqua@staff.univpm.it (M.S.); d.marzioni@univpm.it (D.M.); 2 Clinic of Obstetrics and Gynaecology, Department of Clinical Sciences, Università Politecnica delle Marche, Salesi Hospital, 60123 Ancona, Italy; s.r.giannubilo@staff.univpm.it (S.R.G.); a.ciavattini@univpm.it (A.C.); 3 Department of Life and Environmental Sciences, Università Politecnica delle Marche, 60128 Ancona, Italy; a.frontini@univpm.it

**Keywords:** HTRA1, preeclampsia, pregnancy, HTR8/SVneo, BeWo, cytotrophoblast, syncytiotrophoblast, forskolin

## Abstract

The HtrA serine peptidase 1 (HTRA1) is a multidomain secretory protein with serine–protease activity involved in the regulation of many cellular processes in both physiological and pathological conditions. HTRA1 is normally expressed in the human placenta, and its expression is higher in the first trimester compared to the third trimester, suggesting an important role of this serine protease in the early phases of human placenta development. The aim of this study was to evaluate the functional role of HTRA1 in in vitro models of human placenta in order to define the role of this serine protease in preeclampsia (PE). BeWo and HTR8/SVneo cells expressing HTRA1 were used as syncytiotrophoblast and cytotrophoblast models, respectively. Oxidative stress was induced by treating BeWo and HTR8/SVneo cells with H_2_O_2_ to mimic PE conditions in order to evaluate its effect on HTRA1 expression. In addition, HTRA1 overexpression and silencing experiments were performed to evaluate the effects on syncytialization, cell mobility, and invasion processes. Our main data showed that oxidative stress significantly increased HTRA1 expression in both BeWo and HTR8/SVneo cells. In addition, we demonstrated that HTRA1 has a pivotal role in cell motility and invasion processes. In particular, HTRA1 overexpression increased while HTRA1 silencing decreased cell motility and invasion in HTR8/SVneo cell model. In conclusion, our results suggest an important role of HTRA1 in regulating extravillous cytotrophoblast invasion and motility during the early stage of placentation in the first trimester of gestation, suggesting a key role of this serine protease in PE onset.

## 1. Introduction

The placenta is an essential transitory organ that ensures normal in utero development in humans and mammals [1]. The uterine spiral arteries undergo a physiological change during placentation. In fact, the extravillous trophoblast invades the endometrium until the first third of the myometrium, transforming the placental bed spiral arteries from high-resistance, low-flow vessels to low-resistance and large dilated vessels to ensure an increased blood flow in the intervillous space reducing blood pressure. These alterations of spiral arteries are due to the temporary replacement of the endothelium with a trophoblast layer and to the loss of the smooth muscle cells and elastic lamina present in the wall of these vessels [2,3].

The importance of the development of a normal and functional placenta during pregnancy is highlighted when its development and functions are compromised, then leading to pregnancy complications such as preeclampsia (PE) [4]. PE is a multisystem disorder affecting 5–7% of pregnancies, commonly diagnosed during the second half of pregnancy and clinically characterized by hypertension and proteinuria [5,6]. It has been hypothesized that placental impairment in PE pregnancies is mainly due to poor trophoblast invasion and poor spiral arteries remodeling during the early stage of pregnancy, causing maternal hypertension and a low blood flow to placental villi leading to trophoblast immaturity [7] and endothelial dysfunction [8] making PE a high risk for maternal and neonatal morbidity and mortality [5,6].

The HtrA serine peptidase 1 (HTRA1) is a secreted serine protease belonging to the HTRA1 family proteins [9]. HTRA1 plays a pivotal role in the development of many organs, including the placenta [10,11,12,13,14,15,16]. In fact, HTRA1 expression increases from the first to the third trimester of normal pregnancy [10], and altered HTRA1 expression was found in placentas from gestational trophoblastic diseases [17] and preeclampsia (PE) [18,19]. Moreover, plasma HTRA1 maternal levels were found significantly increased in patients with PE [20], spontaneous preterm birth (sPTB) [21], and gestational diabetes mellitus (GDM) [22]. HTRA1 can degrade extracellular matrix proteins promoting cell migration and invasion [13,15,23]. Thus, HTRA1 may play an essential role in extravillous cytotrophoblast migration in the early stages of pregnancy. Moreover, HTRA1 can regulate TGF-β signaling, a key regulator of angiogenetic processes [24], which are impaired in PE pregnancies [8].

The aim of this study was to evaluate the functional role of HTRA1 in in vitro models of human placenta in order to investigate the possible role of this serine protease in preeclampsia.

## 2. Materials and Methods

### 2.1. Cell Cultures

The human first-trimester trophoblast cell line HTR8/SVneo (a gift by Dr. Charles Graham; Queen’s University, Kingston, ON, Canada) and the human carcinoma cell line BeWo were routinely cultured in the RPMI 1640 medium (Life Technologies, Carlsbad, CA, USA) and in the DMEM/F12 medium (Gibco; Thermo Fisher Scientific, Waltham, MA, USA), respectively. The complete media have been obtained by adding 10% fetal bovine serum (FBS; Gibco) and 100 U/mL penicillin and streptomycin (Gibco). All cell lines used were cultured at 37 °C in an incubator with 5% CO_2_. HTR8/SVneo and BeWo cell lines were used within passages 40 to 50 and 15 to 20, respectively. The morphology of both cell lines was routinely checked by an inverted microscope (Nikon Eclipse Ti, Nikon Instruments, Florence, Italy). Both HTR8/SVneo and BeWo cell lines were routinely tested for E-cadherin and Cytokeratin 7 expression by western blotting analysis. Syncytialization has been induced in BeWo cells by adding Forskolin 50 µM (Santa Cruz Biotechnology, Dallas, TX, USA) for 48 h. Cells treated with DMSO were used as negative control (Vehicle of Foskolin). E-Cadherin was used to validate the syncytialization protocol [25].

### 2.2. Protein Extracts and Western Blotting

HTR8/SVneo and BeWo cells were lysed by using the lysis buffer containing 0.1 M PBS, 0.1% (*w*/*v*) SDS, 1% (*w*/*w*) NONIDET-P40, 1 mM (*w*/*v*) Na orthovanadate, 1 mM (*w*/*w*) PMSF (Phenyl Methane Sulfonyl Fluoride), 12 mM (*w*/*v*) Na deoxycholate, and 1.7 µg/mL Aprotinin, pH 7.5. In order to analyze the intracellular HTRA1 protein, culture media were removed, cells were lysated, centrifuged at 20,000× *g* for 20 min at 4 °C, and the supernatants were aliquoted and stored at −80 °C until use. For the western blotting assay, protein concentration was determined in supernatants previously obtained using the Bradford protein assay (Bio-rad Laboratories, Milan, Italy). Samples were then fractionated by 10% sodium dodecylsulfate (SDS)–polyacrylamide gels and electrophoretically transferred to nitrocellulose membranes. Non-specific protein binding was avoided by incubating the membranes with 5% (*w*/*v*) non-fat dried milk (Biorad Laboratories, Milan, Italy) in TBS/0.05% Tween 20 (TBS-T). Membranes were then incubated overnight at 4 °C with primary antibodies at the appropriate dilution (in TBS-T), as listed in Table 1. After washing, membranes were incubated with the appropriate secondary antibody conjugated with horseradish peroxidase (HRP) (Santa Cruz Biotechnology, Inc., TX, USA) diluted 1:5000 in TBS-T. Antibody binding was detected by the Clarity Western ECL Substrate (Biorad Laboratories, Milan, Italy) using Chemidoc (Biorad Laboratories, Milan, Italy). The densitometric analysis of the obtained bands was performed using ImageJ software (https://imagej.nih.gov/ij/download.html, accessed on 10 November 2022).

### 2.3. Immunofluorescence Staining

HTRA1 expression was evaluated in BeWo and HTR8/SVneo cells by immunofluorescence staining. After washing cells with PBS, cells were fixed with 4% paraformaldehyde diluted in PBS for 10 min at 4 °C and permeabilized with 0.1% Triton X-100 (Sigma, Milano, Italy) in 0.1 M PBS for 5 min at room temperature (RT). After washing in PBS, the non-specific protein binding was avoided by incubating cells with an Animal-Free Blocker (Vector Laboratories, Burlingame, CA, USA) 1:5 in PBS and incubated overnight at 4 °C with rabbit anti-HTRA1 antibody diluted 1:100 (see Table 1). Cells were then washed three times in PBS and incubated with Alexa Fluor 488-conjugated donkey anti-rabbit IgG secondary antibody (Thermo Fisher Scientific) diluted 1:400 in PBS for 30 min at RT. The DAPI probe was used for nuclear staining. Slides were then cover-slipped with a Vectashield mounting medium (Vector Laboratories). Negative controls were obtained by omitting the primary antibody.

### 2.4. Hydrogen Peroxide Treatments

It has been reported that the hydrogen peroxide (H_2_O_2_) concentration in PE pregnancies is 175 µM [26]. Thus, we used 175 µM H_2_O_2_ to induce oxidative stress in HTR8/SVneo and BeWo cells (with and without forskolin) to mimic the PE environment. HTR8/SVneo and BeWo cells (2 × 10^4^ cells/cm^2^) were seeded in six-well plates in the appropriate culture media with or without 175 µM H_2_O_2_ (Sigma-Aldrich) and incubated in the atmosphere with 5% CO_2_ at 37 °C, for 48 h. After treatments, cells were lysed in an ice-cold lysis buffer and centrifuged at 15,000× *g* for 5 min at 4 °C. The supernatants were then aliquoted and stored at −80 °C until use. All experiments were performed in triplicate and were repeated at least three times.

### 2.5. HTRA1 Silencing and Overexpression

HTRA1 silencing and overexpression were performed using a FuGENE HD Transfection Reagent (Promega, Madison, WI, USA), following the manufacturer’s instructions. HTR8/SVneo and BeWo cells were seeded in 6-well plates at 2 × 10^5^ cells/well (for overexpression) or 10^5^ cells/well (for silencing) the day before transfection. Cells were transfected with the pcDNA3.1 (+)-HTRA1 plasmid vector (2 µg/well) for overexpression, while the control cells were transfected with the empty vector (pcDNA3.1 (+) (both vectors from Thermo Fisher Scientific). Cells were transfected with siRNA HTRA1 (2 µM) for silencing, while control cells were transfected with non-targeting negative control siRNA (scramble sequence) (both from Thermo Fisher Scientific). Cells were incubated for 48 h in both procedures, and the efficiency of HTRA1 silencing and overexpression in both cell lines were evaluated by Real-Time PCR and western blot analysis. HTRA1 siRNA (siRNA ID: s11280) sequences were the following: GCCCGUUAGUAAACCUGGAtt (Sense) and UCCAGGUUUACUAACGGGCct (Antisense). PcDNA3.1 (+)-HTRA1 plasmid vector details are reported in Supplementary material S1.

### 2.6. Wound Healing Assay

After transfection with a siRNA HTRA1 or pcDNA3.1 HTRA1 vector, HTR-8/SVneo cells grew to confluence. Then, the cell monolayer was scratch-wounded with a sterile 200 µL pipette tip, and non-adherents were removed by rinsing cells three times with a warm medium. Cells were then incubated at 37 °C with 5% CO_2_ for 48 h. Digital images of the wound were taken at 0, 8, and 24 h at the same position and magnitude. The area of the wound was measured at each time point using ImageJ software (https://imagej.nih.gov/ij/download.html, accessed on 10 November 2022). The experiment was repeated at least three times.

### 2.7. Transwell Invasion Assay

To evaluate the HTR8/SVneo cell invasion capacity after HTRA1 silencing or overexpression, we performed matrigel invasion assays using 24-well transwell inserts (with 8.0 μm pores; cat. no. 353097, Corning, NY, USA) coated with 100 μL of 250 μg/mL LDEV-free Matrigel (cat. no.356234; Corning) diluted in a serum-free RPMI 1640 medium according to the manufacturer’s instructions. After transfection with siRNA HTRA1 or pcDNA3.1 HTRA1 plasmid, HTR8/SVneo cells (5 × 10^4^) were resuspended in serum-free RPMI 1640 medium and plated into the upper chambers of transwell plates on top of the Matrigel. The lower chambers were filled with 750 μL of an RPMI 1640 medium supplemented with a chemoattractant (10% FBS). After 24 h of culture, non-invading cells remained in the upper chamber, and Matrigel was gently removed from the apical side of the membrane using a cotton swab. The migrated cells remaining on the lower surface of the membrane were fixed with 100% methanol for 20 min and stained with 0.5% crystal violet dye for 15 min at RT. Stained cells were counted by capturing four non-overlapping fields of view per sample at 10 × magnification using ImageJ software (https://imagej.nih.gov/ij/download.html, accessed on 10 November 2022). Data were expressed as mean ± standard deviation (SD) of invaded cells. The experiments were repeated three times in triplicate.

### 2.8. Statistical Analysis

Quantitative variables were summarized using quartiles, and a Wilcoxon–Mann–Whitney test was performed to compare groups. Results were graphically represented using bar plots.

## 3. Results

### 3.1. HTRA1 Protein Is Expressed in Placental Cell Lines

**Figure 1:**
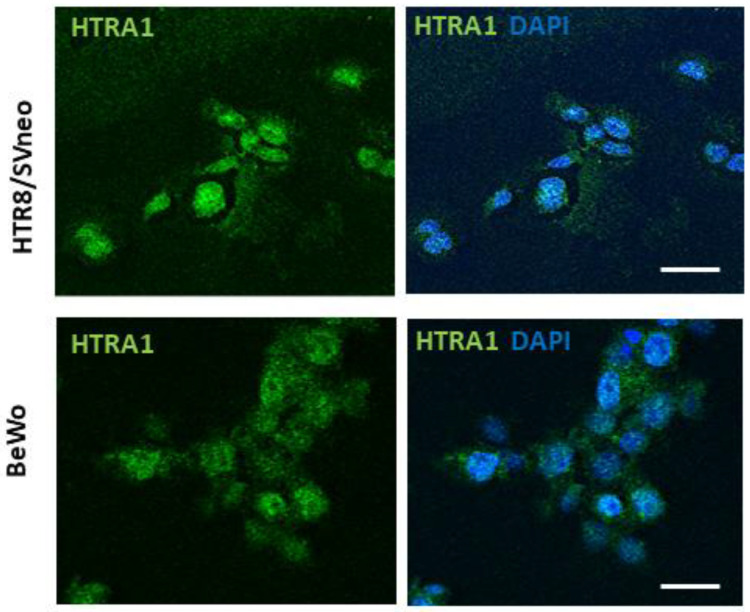
HTRA1 expression in human placenta cell lines. Immunofluorescence showing HTRA1 (green staining) expression in the cytoplasm of HTR8/SVneo and BeWo cell lines. Nuclei are stained in blue (DAPI probe). Scale bars = 50 µm.

In order to investigate HTRA1 protein expression in BeWo and HTR8/SVneo cell lines, we evaluated HTRA1 cellular expression and localization by immunofluorescence. As shown in Figure 1, both cell lines showed a cytoplasmic expression of the HTRA1 protein. Thus, both cell lines analyzed are suitable as models to study the pathophysiological role of HTRA1 in human pregnancy.

### 3.2. Oxidative Stress Increased HTRA1 Protein Expression in BeWo and HTR8/SVneo Cells

**Figure 2:**
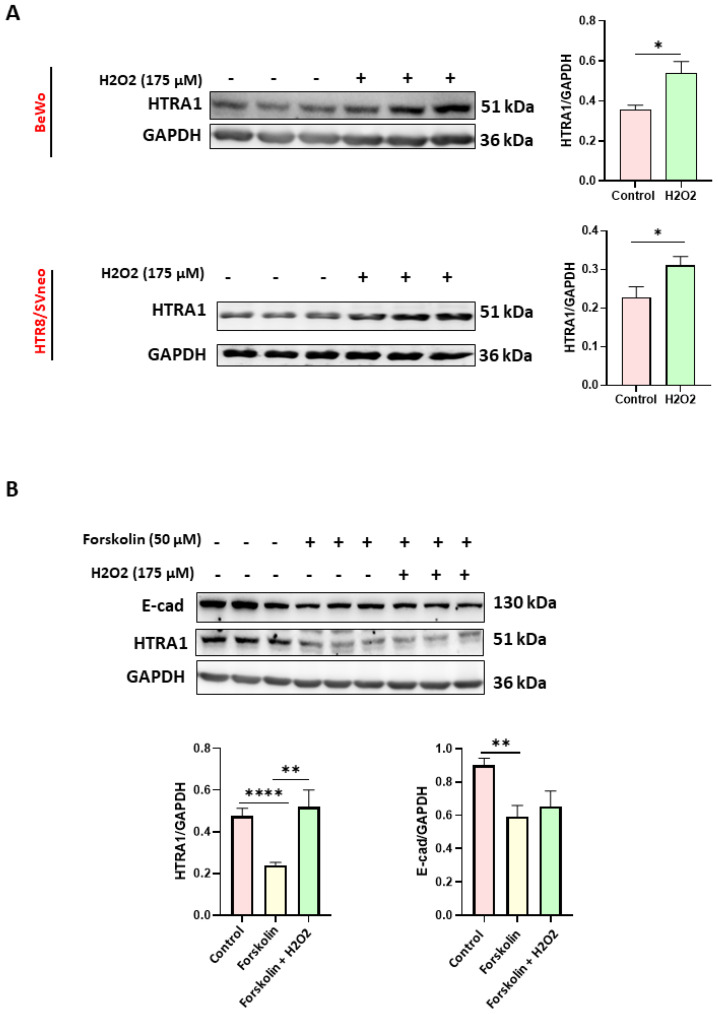
Effect of oxidative stress on HTRA1 protein expression in both BeWo and HTR8/SVneo cells. H_2_O_2_ exposure significantly increases HTRA1 protein expression in both cell lines (**A**). Forskolin was used to induce BeWo cell syncytialization (**B**). Bands of western blotting were quantified, and the results were calculated in arbitrary units (AU) and reported as bars of a histogram to the right of the representative western blotting. Data are represented as mean ± SD. * *p* < 0.05, ** *p* < 0.01, and **** *p* < 0.0001.

Since it has been proven that oxidative stress is a characteristic feature of preeclamptic pregnancies [26], we evaluated the effect of oxidative stress on HTRA1 expression in HTR8/SVneo and BeWo cell lines; two placental cell lines were used as models of extravillous [27,28,29] and villous cytotrophoblast [30,31,32], respectively. Moreover, the BeWo cell line can be used as a syncytiotrophoblast model when syncytialization is induced by forskolin [25]. As shown in Figure 2, HTRA1 expression was significantly increased under oxidative stress in both BeWo and HTR8/SVneo cells (Figure 2A). In addition, HTRA1 expression was significantly lower when BeWo cells were syncytialized (treated only with forskolin without H_2_O_2_), while oxidative stress, induced by H_2_O_2_, significantly increased HTRA1 expression (Figure 2B), demonstrating that the oxidative stress can modulate HTRA1 expression in preeclamptic placenta.

### 3.3. HTRA1 mRNA and Protein Expression Were Efficiently Modulated under Overexpressing and Silencing Conditions

**Figure 3:**
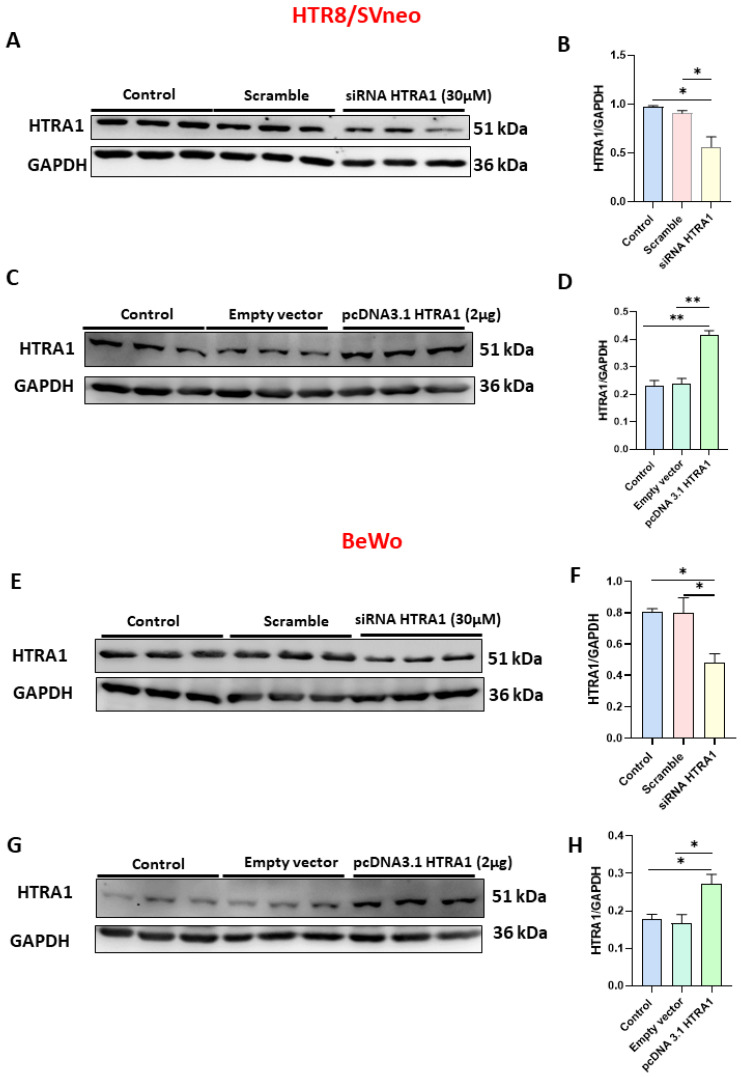
Validation of HTRA1 overexpression (by pcDNA3.1 HTRA1 plasmid) and silencing (by siRNA HTRA1) in HTR8/SVneo (**A**–**D**) and BeWo (**E**–**H**) cell lines. HTRA1 protein quantifications in HTR8/SVneo cells (**B**,**D**) and BeWo cells (**F**,**H**) are reported as bars of the histogram. HTRA1 representative western blots for HTR8/SVneo cells are depicted in (**A**,**C**); for BeWo, cells are depicted in (**E**,**G**). The results were calculated in arbitrary units (AU). Data are represented as mean ± SD. * *p* < 0.05 and ** *p* < 0.01.

In order to investigate the functional role of HTRA1 in HTR8/SVneo and BeWo cell lines, we overexpressed (by pcDNA3.1 plasmid) and silenced (by siRNA) HTRA1 expression in both cell lines. As shown in Figure 3, when HTRA1 was silenced by a specific siRNA sequence, HTRA1 protein expression was significantly decreased in HTR8/SVneo (Figure 3A,B) and BeWo (Figure 3E,F) cells compared to the control and scramble sequence. Contrarily, when HTRA1 was overexpressed by pcDNA3.1 plasmid, HTRA1 protein expression was significantly increased in HTR8/SVneo (Figure 3C,D) and BeWo (Figure 3G,H) cells compared to the control and empty vector. Then, HTRA1 expression was efficiently modulated under silencing and overexpressing conditions.

### 3.4. HTRA1 Protein Overexpression and Silencing Did Not Alter BeWo Syncytialization

**Figure 4:**
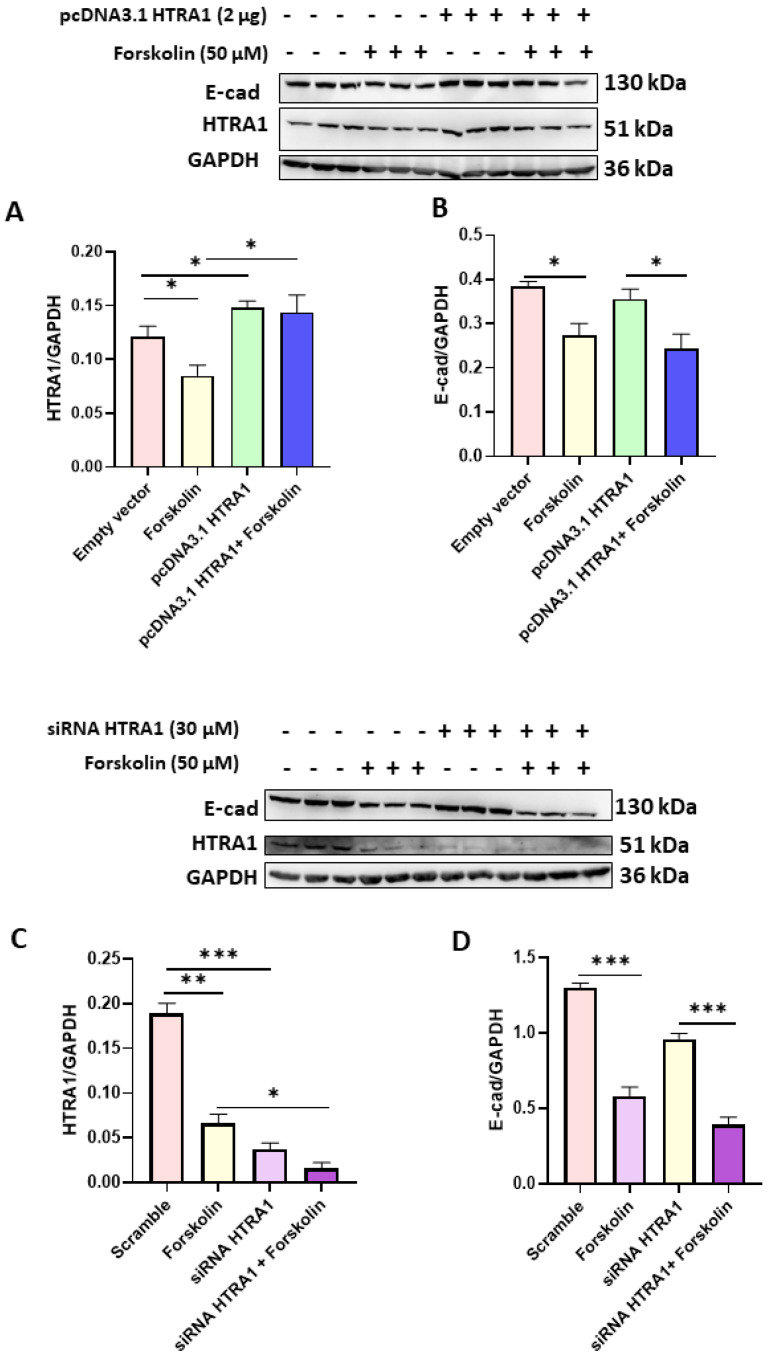
Effects of HTRA1 overexpression (**A**,**B**) and silencing (**C**,**D**) in syncyzialized BeWo cells by forskolin. Two representative western blots are reported for overexpression and silencing experiments. The bands were quantified, and the results were calculated in arbitrary units (AU) and reported as bars of a histogram. Data are represented as mean ± SD. * *p* < 0.05, ** *p* < 0.01 and *** *p* < 0.001.

Since we found that HTRA1 expression was decreased in BeWo syncyzialized cells (see Figure 2A), we investigated if the syncytialization process could be modulated by HTRA1 in these cells. As reported in Figure 4A,C, both HTRA1 overexpression and silencing significantly modulated HTRA1 expression, but they did not alter the syncytialization process as shown by E-Cadherin expression (Figure 4B,D). HTRA1 overexpression and silencing did not alter E-Cadherin expression if compared with control (empty vector or scramble), while E-Cadherin expression was altered if treated with forskolin, demonstrating that the syncytialization process is independent of HTRA1.

### 3.5. HTRA1 Protein Regulates HTR8/SVneo Cells Motility

**Figure 5:**
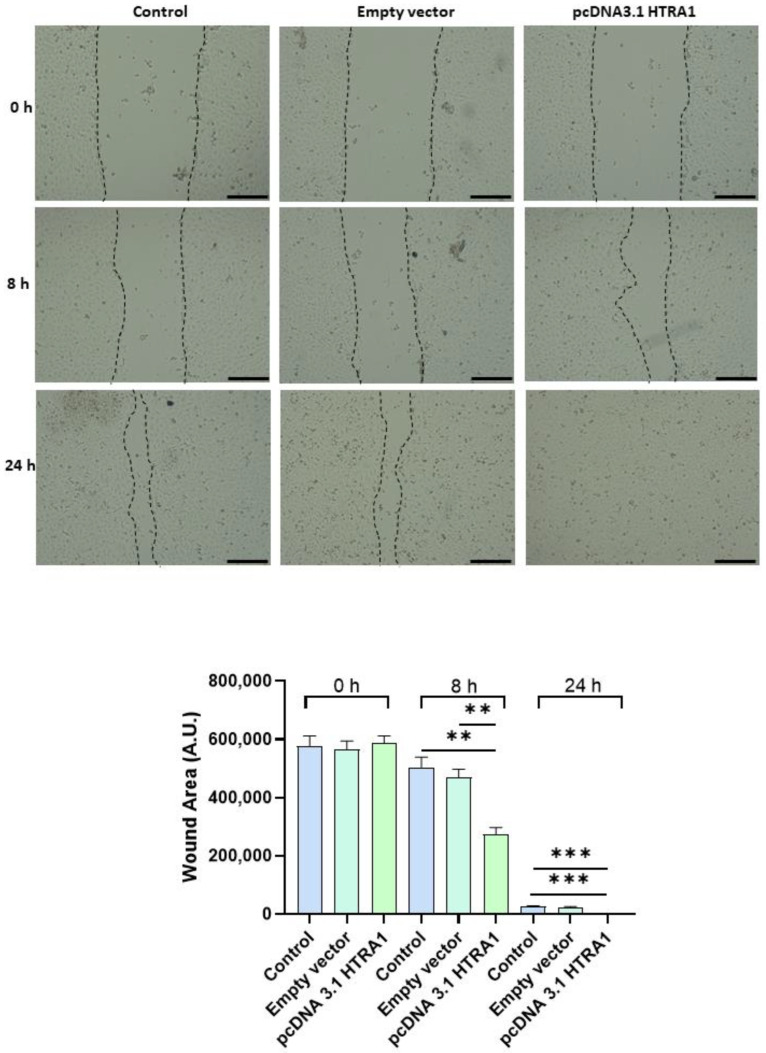
Wound healing assay after HTRA1 overexpression in HTR8/SVneo cells. Representative photos of wound areas are shown. Wound areas were expressed in arbitrary units (AU) and reported as bars of a histogram. Data are represented as mean ± SD. ** *p* < 0.01 and *** *p* < 0.001. Scale bar: 200 µm.

**Figure 6:**
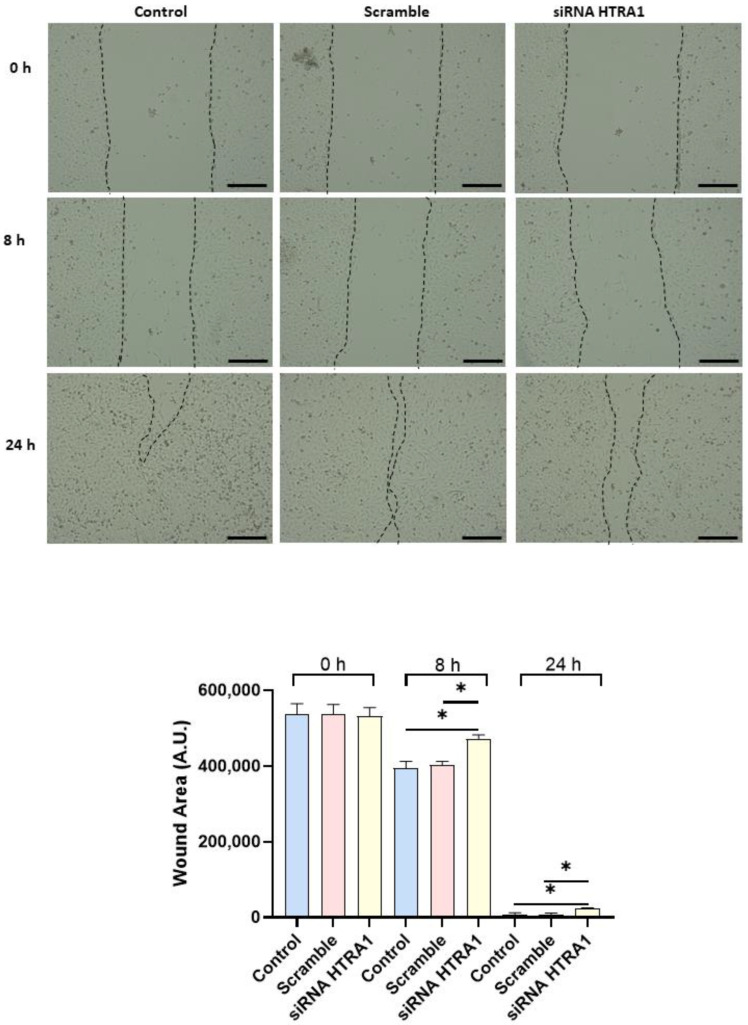
Wound healing assay after HTRA1 silencing in HTR8/SVneo cells. Representative photos of wound areas are shown. Wound areas were expressed in arbitrary units (AU) and reported as bars of a histogram. Data are represented as mean ± SD. * *p* < 0.05. Scale bar: 200 µm.

Since cell migration plays a key role in the placental formation, we investigated if HTRA1 overexpression and silencing could alter HTR8/SVneo cell motility. As shown in Figure 5, HTRA1 overexpression by pcDNA3.1 plasmid significantly improved HTR8/SVneo cell motility; note that the wound was completely closed compared to the 24 h control and empty vector, which showed an unclosed area. HTRA1 silencing by siRNA sequence significantly impaired the motility of these cells (Figure 6); note that HTRA1 silencing significantly slowed wound closure compared to the control and scramble sequence. Thus, we can state that HTRA1 plays a key role in regulating the motility of extravillous cytotrophoblast cells.

### 3.6. HTRA1 Protein Regulates HTR8/SVneo Cells Invasion

**Figure 7:**
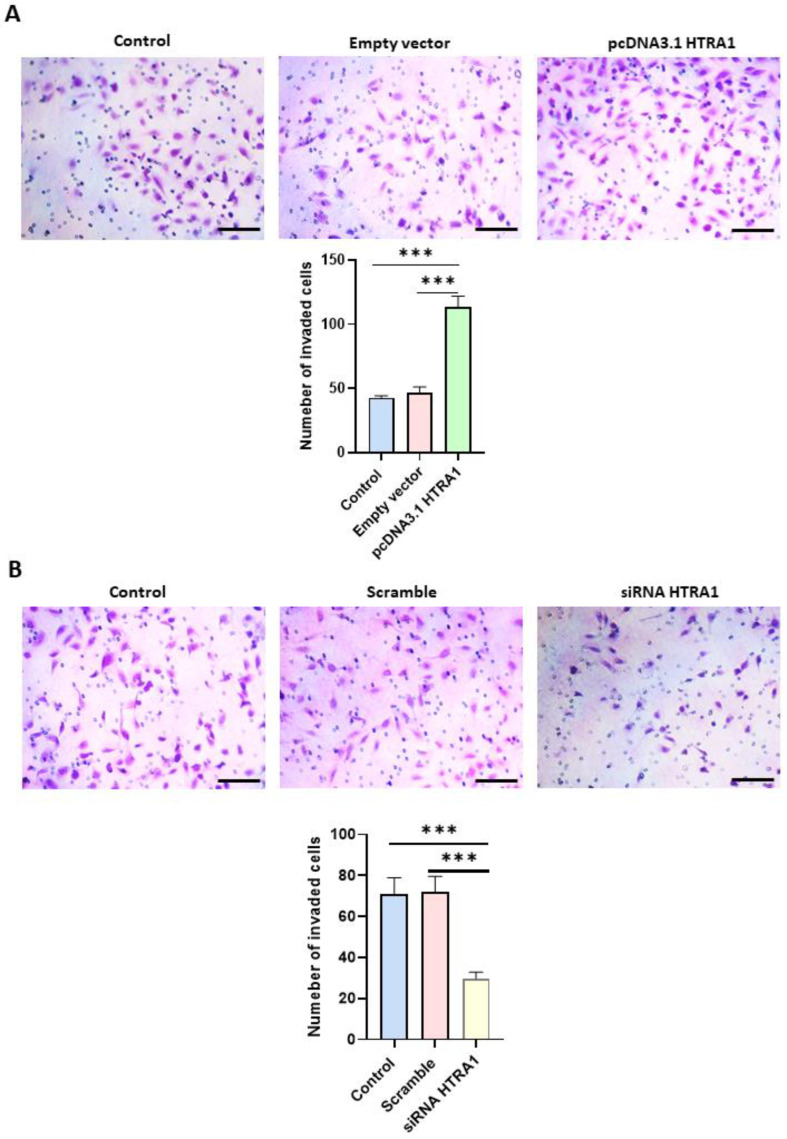
Matrigel invasion assay after HTRA1 overexpression (**A**) and silencing (**B**) in HTR8/SVneo cells. The images show cells invading Matrigel. Results are expressed as the number of invading cells and reported as bars of a histogram. Data are represented as mean ± SD. *** *p* < 0.001. Scale bar: 40 µm.

Cell migration is an essential process in normal placental development, and a shallow invasion of the extravillous cytotrophoblast leads to PE onset [32]. Since HTRA1 is a serine protease that can degrade the extracellular matrix component such as fibronectin [13], we investigated if HTRA1 overexpression and silencing could alter HTR8/SVneo cell invasion by using Matrigel invasion assay. As shown in Figure 7A, HTRA1 overexpression by pcDNA3.1 plasmid significantly facilitated cell invasion compared to the control and empty vector. Contrarily, HTRA1 silencing by siRNA significantly reduced cell invasion compared to the control and scramble sequence (Figure 7B). Thus, HTRA1 protein expression can significantly modulate the invasion ability of extravillous cytotrophoblast cells.

## 4. Discussion

HTRA1 is a secreted protein normally expressed in the placenta from the first to third trimester [17]. Moreover, HTRA1 expression is altered in PE pregnancies in both placental tissue and maternal blood [33]. As previously demonstrated, HTRA1 is mainly expressed in the villous cytotrophoblast in the first trimester of gestation, in both syncytium and villous cytotrophoblast in the second trimester, and mainly in the syncytium in the third trimester of pregnancy [17]. In our study, we showed that HTRA1 protein was expressed in the cytoplasm of BeWo and HTR8/SVneo cells. Both these cells are good models for studying the role of HTRA1 in placental pathophysiology. Interestingly, we found that HTRA1 expression significantly decreased during BeWo syncytialization, proving that HTRA1 expression is lost during the syncytialization process, suggesting a prevalent role in processes related to cell invasiveness or proliferation characteristic of cytotrophoblast cells but not of syncytiotrophoblast.

In fact, HTRA1 overexpression and silencing in BeWo cells did not alter the syncytialization process in these cells, suggesting that the loss of HTRA1 is a consequence and not the cause of this process, proving that HTRA1 does not play an active role in the syncytialization process. Presumably, this occurs during the first trimester of pregnancy, where HTRA1 is expressed only in the villous cytotrophoblast, while the syncytium is negative for HTRA1 in the first-trimester placenta [10]. It is known that preeclamptic pregnancies are characterized by the presence of oxidative stress [26], an important process also reported in many other pathologies, including cancer [34,35]. Moreover, it has been reported that oxidative stress plays a key role in promoting the inflammation process [36,37]. Interestingly, we found that inducing oxidative stress in vitro significantly increased HTRA1 expression in both BeWo and HTR8/SVneo cell lines. These data suggest that HTRA1 may be increased in the first trimester placentas during PE onset, reflecting the HTRA1 plasma level increase in the first trimester of pregnancy in women who will later develop PE. Extravillous cytotrophoblast motility and invasion play an essential role in the development of a normal and functional placenta in the early stage of placentation, and an alteration of this process can lead to a shallow invasion causing PE onset [38]. HTRA1 is a protease that can degrade extracellular matrix, promoting cell motility and invasion. The role of HTRA1 in HTR8/SVneo cell invasion and motility is still a matter of debate. In fact, few studies reported a decreased migration and invasion when HTRA1 was up-regulated by specific plasmids [18,39,40]. Another study reported a significant increase in HTR8/SVneo cell invasion when HTRA1 was overexpressed [41]. Our results are in agreement with the latter study. In fact, overexpression of HTRA1 significantly improved cell motility and invasion processes proving the important role of this serine protease. In addition, further validation of the HTRA1 function was demonstrated by our experiments on HTRA1 silencing, which showed a significant decrease in HTR8/SVneo cell motility and invasion. Thus, this serine protease may be involved in regulating motility and invasion of the extravillous cytotrophoblast.

Since this is an in vitro study, its limitation consists of the impossibility of validating our results in human placental samples at the first trimester of gestation. In fact, it is not possible to recruit these samples since PE onset occurs after the 20th week of gestation. Thus, it is impossible to know the expression levels of HTRA1 in the placenta affected by PE in the first trimester of gestation.

## 5. Conclusions

In conclusion, we can state that HTRA1 is overexpressed in oxidative stress condition that is characteristic of PE pregnancies. In addition, its modulation contributes to cell motility and invasion, suggesting an important role in placenta development. Although it is impossible to know the HTRA1 expression levels in placental tissues in the first trimester that will develop PE later in pregnancy, we proved that HTRA1 plays an important role in extravillous trophoblast cell motility, allowing us to hypothesize that an alteration of HTRA1 expression can contribute, at least in part, to PE onset.

## Figures and Tables

**Table 1:** Antibodies used in this study.

Antibody	Western Blot Dilution	Company
pAb Rabbit anti-human HTRA1 (#PA5-11412)	1:500	Thermo Fisher Scientific, Waltham, US
mAb Mouse anti-human E-cadherin (#sc-8426)	1:400	Santa Cruz Biotechnology, Dallas, US
mAb Mouse anti-human Cytokeratin 7 (#sc-23876)	1:400	Santa Cruz Biotechnology, Dallas, US
mAb Mouse anti-human GAPDH (#sc- 47724)	1:1000	Santa Cruz Biotechnology, Dallas, US

mAb: monoclonal antibody; pAb: polyclonal antibody.

## Data Availability

The data presented in this study are available upon request from the corresponding author.

## Supplementary Materials

The following supporting information can be downloaded at: https://www.mdpi.com/article/10.3390/cimb45050246/s1, Supplementary File S1: PcDNA3.1 (+)-HTRA1 plasmid vector details.
