# Supplementary material for: HTRA1 in Placental Cell Models: A Possible Role in Preeclampsia
Source: Curr Issues Mol Biol. 2023 May 1;45(5):3815–28. doi: 10.3390/cimb45050246 (PMC10216960; doi:10.3390/cimb45050246)
Supplement: Supplementary file 1 [file cimb-45-00246-s001.zip › Supplementary File S1.pdf]

Sequence name: HTRA1

Sequence type: DNA

Biosafety level: Level 1

Express cloning vector / RS: pcDNA3.1(+), NheI/NotI

TSE free: No

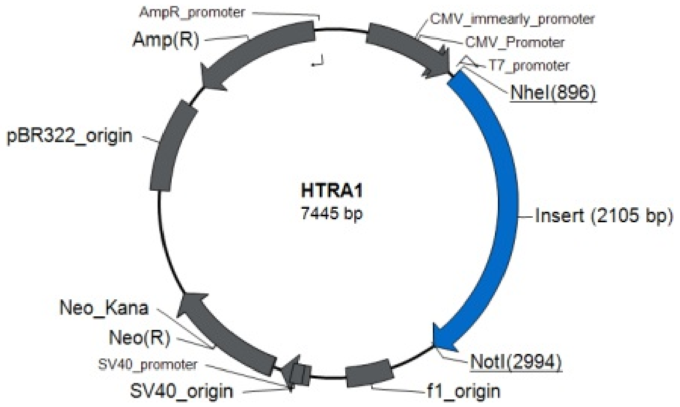

Sequence name / optimized for

HTRA1/ Homo sapiens

ORF	Protected sites	Protected areas	Motifs to avoid
107-1549 [ATG...TGA]	1-6 NheI [GCTAGC]<br>2098-2105 NotI [GCGGCCGC]		NheI [GCTAGC]<br>NotI [GCGGCCGC]

```
1.    GCTAGCACTCGCACCCGCTGCCCCCGAGGCCCTCCTGCACTCTCCCCGGCGCCGCTCTCCGGCCCTCGC
                                            M  Q  I  P  R  A  A  L  L  P  L
70.   CCTGTCCGCCGCCACCGCCGCCGCCGCCAGAGTCGCCATGCAAATTCCTAGAGCTGCCCTGCTGCCTCT
      L  L  L  L  L  A  A  P  A  S  A  Q  L  S  R  A  G  R  S  A  P  L  A
139.  CCTGCTGTTGCTTCTTGCTGCCCCTGCTTCTGCCCAGCTGTCTAGAGCTGGAAGATCTGCCCCTCTGGC
      A  G  C  P  D  R  C  E  P  A  R  C  P  P  Q  P  E  H  C  E  G  G  R
208.  CGCTGGCTGTCCTGATAGATGTGAACCCGCCAGATGTCCTCCTCAGCCTGAGCATTGTGAAGGCGGCAG
      A  R  D  A  C  G  C  C  E  V  C  G  A  P  E  G  A  A  C  G  L  Q  E
277.  AGCCAGAGATGCCTGCGGCTGTTGTGAAGTGTGCGGAGCACCTGAAGGCGCCGCTTGTGGACTTCAAGA
      G  P  C  G  E  G  L  Q  C  V  V  P  F  G  V  P  A  S  A  T  V  R  R
346.  AGGACCTTGCGGAGAGGGCCTGCAGTGCGTTGTGCCTTTTGGAGTGCCTGCCTCTGCCACAGTTAGGCG
      R  A  Q  A  G  L  C  V  C  A  S  S  E  P  V  C  G  S  D  A  N  T  Y
415.  GAGAGCACAAGCTGGCCTGTGTGTGTGCGCCTCTTCTGAGCCAGTGTGTGGCTCCGACGCCAACACCTA
      A  N  L  C  Q  L  R  A  A  S  R  R  S  E  R  L  H  R  P  P  V  I  V
484.  CGCCAATCTGTGTCAGCTGAGAGCCGCCAGCAGAAGAAGCGAGAGACTGCACAGACCTCCAGTGATCGT
      L  Q  R  G  A  C  G  Q  G  Q  E  D  P  N  S  L  R  H  K  Y  N  F  I
553.  GCTGCAGAGAGGCGCTTGCGGACAAGGCCAAGAGGACCCCAATAGCCTGCGGCACAAGTACAACTTTAT
      A  D  V  V  E  K  I  A  P  A  V  V  H  I  E  L  F  R  K  L  P  F  S
622.  CGCCGACGTGGTGGAAAAGATCGCCCCTGCCGTGGTGCACATCGAGCTGTTCAGAAAGCTGCCCTTCAG
      K  R  E  V  P  V  A  S  G  S  G  F  I  V  S  E  D  G  L  I  V  T  N
691.  CAAGCGCGAAGTGCCTGTGGCTTCTGGCAGCGGCTTTATCGTGTCTGAGGACGGCCTGATCGTGACAAA
      A  H  V  V  T  N  K  H  R  V  K  V  E  L  K  N  G  A  T  Y  E  A  K
760.  CGCCCACGTGGTCACCAACAAGCACAGAGTGAAGGTGGAACTGAAGAACGGCGCCACCTACGAGGCCAA
      I  K  D  V  D  E  K  A  D  I  A  L  I  K  I  D  H  Q  G  K  L  P  V
829.  GATCAAGGACGTGGACGAGAAGGCCGATATCGCCCTGATCAAGATCGACCACCAGGGCAAGCTGCCAGT
      L  L  L  G  R  S  S  E  L  R  P  G  E  F  V  V  A  I  G  S  P  F  S
898.  GCTGCTGTTGGGAAGAAGCAGCGAACTGAGGCCTGGCGAGTTTGTGGTGGCCATCGGATCTCCATTCAG
      L  Q  N  T  V  T  T  G  I  V  S  T  T  Q  R  G  G  K  E  L  G  L  R
967.  CCTGCAGAACACCGTGACCACCGGCATCGTGTCCACAACACAAAGAGGCGGCAAAGAGCTGGGCCTGAG
      N  S  D  M  D  Y  I  Q  T  D  A  I  I  N  Y  G  N  S  G  G  P  L  V
1036. AAACAGCGACATGGACTACATCCAGACCGACGCCATCATCAACTACGGCAACAGCGGCGGACCCCTGGT
      N  L  D  G  E  V  I  G  I  N  T  L  K  V  T  A  G  I  S  F  A  I  P
1105. CAATCTGGATGGCGAAGTGATCGGCATCAACACCCTGAAAGTGACAGCCGGCATCAGCTTCGCTATCCC
      S  D  K  I  K  K  F  L  T  E  S  H  D  R  Q  A  K  G  K  A  I  T  K
1174. [illegible]
```

1174. CAGCGATAAGATCAAGAAGTTCCTGACCGAGAGCCACGACCGGCAGGCCAAGGGAAAAGCCATCACCAA

K K Y I G I R M M S L T S S K A K E L K D R H

1243. GAAGAAGTACATCGGAATCCGGATGATGAGCCTGACCAGCAGCAAGGCCAAAGAACTGAAGGACCGGCA

R D F P D V I S G A Y I I E V I P D T P A E A

1312. CAGAGACTTCCCCGATGTGATCTCTGGCGCCTACATCATTGAAGTGATCCCCGACACACCAGCCGAAGC

G G L K E N D V I I S I N G Q S V V S A N D V

1381. CGGCGGACTGAAAGAAAACGACGTGATCATCAGCATCAACGGCCAGAGCGTGGTGTCCGCCAACGATGT

S D V I K R E S T L N M V V R R G N E D I M I

1450. GTCTGATGTGATCAAGCGCGAGAGCACCCTGAACATGGTTGTGCGGAGGGGCAACGAGGACATCATGAT

T V I P E E I D P *

1519. CACCGTGATTCCCGAGGAAATCGACCCCTGAGCAGAGGCATGAGCTGGACTTCATGTTTCCCTCAAAGA

1588. CTCTCCCGTGGATGACGGATGAGGACTCTGGGCTGCTGGAATAGGACACTCAAGACTTTTGACTGCCAT

1657. TTTGTTTGTTCAGTGGAGACTCCCTGGCCAACAGAATCCTTCTTGATAGTTTGCAGGCAAAACAAATGT

1726. AATGTTGCAGATCCGCAGGCAGAAGCTCTGCCCTTCTGTATCCTATGTATGCAGTGTGCTTTTTCTTGC

1795. CAGCTTGGGCCATTCTTGCTTAGACAGTCAGCATTTGTCTCCTCCTTTAACTGAGTCATCATCTTAGTC

1864. CAACTAATGCAGTCGATACAATGCGTAGATAGAAGAAGCCCCACGGGAGCCAGGATGGGACTGGTCGTG

1933. TTTGTGCTTTTCTCCAAGTCAGCACCCAAAGGTCAATGCACAGAGACCCCGGGTGGGTGAGCGCTGGCT

2002. TCTCAAACGGCCGAAGTTGCCTCTTTTAGGAATCTCTTTGGAATTGGGAGCACGATGACTCTGAGTTTG

2071. AGCTATTAAAGTACTTCTTACACATTGGCGGCCGC
